# Effects of Sample Size on Estimates of Population Growth Rates Calculated with Matrix Models

**DOI:** 10.1371/journal.pone.0003080

**Published:** 2008-08-28

**Authors:** Ian J. Fiske, Emilio M. Bruna, Benjamin M. Bolker

**Affiliations:** 1 Department of Wildlife Ecology & Conservation, University of Florida, Gainesville, Florida, United States of America; 2 Center for Latin American Studies, University of Florida, Gainesville, Florida, United States of America; 3 Biological Dynamics of Forest Fragments Project, Instituto Nacional de Pesquisas da Amazônia and Smithsonian Tropical Research Institute C.P. 478, Manaus, Brazil; 4 Department of Zoology, University of Florida, Gainesville, Florida, United States of America; University of Sheffield, United Kingdom

## Abstract

**Background:**

Matrix models are widely used to study the dynamics and demography of populations. An important but overlooked issue is how the number of individuals sampled influences estimates of the population growth rate (λ) calculated with matrix models. Even unbiased estimates of vital rates do not ensure unbiased estimates of λ–Jensen's Inequality implies that even when the estimates of the vital rates are accurate, small sample sizes lead to biased estimates of λ due to increased sampling variance. We investigated if sampling variability and the distribution of sampling effort among size classes lead to biases in estimates of λ.

**Methodology/Principal Findings:**

Using data from a long-term field study of plant demography, we simulated the effects of sampling variance by drawing vital rates and calculating λ for increasingly larger populations drawn from a total population of 3842 plants. We then compared these estimates of λ with those based on the entire population and calculated the resulting bias. Finally, we conducted a review of the literature to determine the sample sizes typically used when parameterizing matrix models used to study plant demography.

**Conclusions/Significance:**

We found significant bias at small sample sizes when survival was low (survival = 0.5), and that sampling with a more-realistic inverse J-shaped population structure exacerbated this bias. However our simulations also demonstrate that these biases rapidly become negligible with increasing sample sizes or as survival increases. For many of the sample sizes used in demographic studies, matrix models are probably robust to the biases resulting from sampling variance of vital rates. However, this conclusion may depend on the structure of populations or the distribution of sampling effort in ways that are unexplored. We suggest more intensive sampling of populations when individual survival is low and greater sampling of stages with high elasticities.

## Introduction

Matrix models [1], [2] are an important tool used by ecologists to study the demography of structured populations and for conducting population viability analyses. They are flexible, readily applicable to a diversity of life-history strategies, and there is a broad body of literature describing their construction, interpretation, and limitation reviewed in [3], [4]. However as a recent review by Doak et al. [5] cogently summarizes, they are data-hungry models requiring detailed estimates of birth, death, reproduction, and other vital rates. When the number of individuals used to estimate vital rates is low, the resulting vital rates–as well as estimates of variances and covariances among them–can be biased. Because biased vital rates can lead to inaccurate projections of the population growth rate (i.e., λ), there has been an upsurge in studies exploring alternative sampling designs for demographic studies [5]–[7].

Nevertheless, even unbiased estimates of vital rates do not ensure unbiased estimates of the population growth rate. This is because λ is the dominant eigenvalue of the transition matrix [2], and hence a nonlinear function of the underlying vital rates (i.e., *λˆ* = *f*(*v*_1_,*v*_2_,…,*v_n_*), where the *v_i_* are the *n* vital rates and *f* is a nonlinear real-valued function). As for other nonlinear functions describing ecological processes e.g., [8], [9], the mathematical theorem known as Jensen's inequality [10] implies that variance in vital rates–even those that have been accurately measured–will bias estimates of λ. The amount and direction of this bias depend both on the strength of the nonlinearity of the relationship between λ and the vital rates, and on the variance of the vital rates themselves.

Variance in vital rates can arise from two sources. The first of these is *process variance*, which results from real variation in the population over space or time [11]–[13]. The second source is *sampling variance*, which is a result of studying a sample rather than the entire population. Several studies have used Jensen's Inequality to predict potential biases in λ resulting from process variance e.g., [14], [15], and methods for dealing with process variance, especially over time, are well-developed [16]. While there are also methods that attempt to separate sampling variance from the total observed variance in vital rates [17], [18], the potential for sampling variance to bias estimates in λ has received limited attention. Houllier et al. [19] used analytical approaches and stochastic simulations to test for biases resulting from variance of the matrix elements, while Usher [20] derived analytic solutions for both Leslie matrices and more general models. In general, these studies found that biases in estimates of λ were small–usually less than 0.5%. However, the potential for sampling variance to bias estimates of λ has yet to be investigated for matrix models in which organisms are capable of regressing into smaller size classes. These models are extremely common–they represent the demography of organisms ranging from plants to marine invertebrates e.g., [12], [21].

Jensen's Inequality would lead one to predict that even when the estimates of the vital rates are accurate, small sample sizes will lead to biased estimates of λ as a result of increased sampling variance. To understand why, one must first understand how this variance is modeled in demographic studies. Lower-level vital rates sensu [4] are often modeled as binomial random variables. The binomial distribution, which assumes homogeneous vital rates among individuals within a stage class, has a higher variance for a given mean value than a model with heterogeneous vital rates [22]. Therefore, the binomial distribution is a conservative model for the sampling process. The sampling variance of these binomial vital rates is:
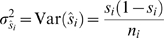
(1)where *n_i_* is the number of individuals in class *i* in the sample, and *s_i_* is the true value of the vital rate [23]. Therefore, sampling variation of a binomial vital rate is maximized when the true rate is equal to 0.5. For estimates of fecundity, such as the number of offspring an individual produces given that it reproduces at all, the variance is:
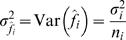
(2)where *f_i_* is the fecundity of individuals in class *i* and *σ_i_* ^2^ is the true variance of the fecundity among individuals in class *i*; *n_i_* is defined as above. Thus, as the variation of the true fecundity increases, so does the variation of the estimated fecundity. Furthermore, as sample size decreases these sample variances increase. This has the net effect of biasing estimates of λ.

We used stochastic simulations to determine if sampling variance biased estimates of λ and how these biases varied with the sample size used to construct matrix models. Because the distribution of individuals among a population's size classes has been shown to influence the outcome of demographic analysis [5]–[7], we also considered the potential for synergistic effects of sample size and population structure on estimates of λ and potential biases in these estimates. Our simulations were conducted using multi-year demographic data collected to elucidate the population dynamics of Amazonian understory herb *Heliconia acuminata* (Heliconiaceae) [24], [25]. Using annual transition matrices constructed with six years of demographic data from 3842 plants, we addressed three questions. First, does the sampling variance of two key vital rates, survival and fecundity, bias estimates of population growth rates? To address this question, we compared the “true” growth rate of the total study population (hereafter, λ) with the growth rates of subpopulations composed of 25–200 randomly selected individuals (hereafter, *λˆ*). Second, are the patterns of bias influenced by population structure? We conducted our simulations with two different population structures. First, we used a uniform distribution of individuals among size classes (i.e., equal numbers of individuals in all size classes), which has been put forward as the optimal sampling distribution for demographic studies [7]. We also used a distribution that reflects the biological structure of many populations in the field, known as the “inverse J distribution” [24], [26]. An “inverse J distribution” contains fewer stage *i*+1 individuals than stage *i*, such that a histogram of stage classes in a sample is a reflected “J” shape. Finally, what range of sample sizes is typically used to parameterize matrix models of plant demography, and how do these sample sizes compare with those at which bias in estimates of λ becomes negligible?

## Results

### Does the sampling variance of two key vital rates, survival and fecundity, bias estimates of λ?

**Figure 1:**
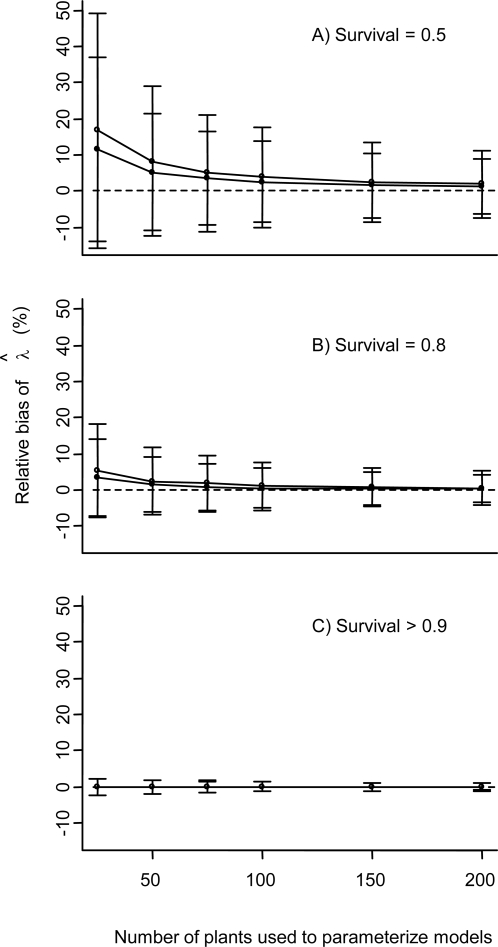
Relative bias in estimates of λ (±1 SD) with increasing sample sizes and (A) survival = 0.5, (B) survival = 0.8, and (C) survival = 0.9. Bias is calculated using the equation (λˆ−λ)/λ×100%. Results are shown for uniform sampling of all stage classes (filled symbols) and sampling from a more realistic J-distribution (open symbols). Sample sizes on the abscissa are the total number of plants (summed across all stage classes) used for parameterizing matrix models. The dashed line indicates a bias = 0.

As the sample sizes used to calculate vital rates decreased, *λˆ* increasingly overestimated λ (maximum bias = 16.61%±32.4 SD; Figure 1). This maximum bias occurred when simulating an inverse-J sampling distribution with 25 individuals and 0.5 survival probability. However, the observed bias became negligible as the rates of individual survival or sample sizes increased. For instance, using 50 individuals to estimate vital rates from a population with a mean survival probability of 0.5 resulted in a mean bias of 6.61%±18.65 SD (Figure 1a), while increasing the survival rate to 0.8 resulted in a mean bias of only 1.88%±8.55 SD (Figure 1b). The coefficient of variation (CV) of fecundity, which increased up to 32-fold in the different scenarios we modeled, did not bias λˆ in any of our simulations.

### Are the patterns of bias influenced by population structure?

The amount of bias increased when simulations were run with a more realistic inverse-J population structure than when using equal numbers of individuals in all stage classes (8.08%±20.70 SD vs. 5.14%±16.22 SD respectively when sampling 50 individuals when survival = 0.5; 2.33%±9.32 SD vs. 1.43%±7.67 SD when survival = 0.8; Figure 1). This result was qualitatively similar for all combinations of survival and sample size.

### What sample sizes are used to parameterize matrix models of plant demography, and how do these compare with those at which bias in estimates of λ becomes negligible?

Our literature review resulted in 28 studies of perennial herbs, 16 of trees, 9 of shrubs, and 15 studies of other plant types (e.g., grasses, geophytes, forbs; Appendix S1). Of these 68 studies, however, we were only able to determine the number of plants that had been used to parameterize the matrix models in 52 (Table 1). Studies of perennial herbs used fewer individuals than those of trees. Approximately 12% of the studies on perennial herbs used fewer than 100 individuals (summed across all stage classes), and only 25% of studies were based on 500 or more individuals (range: 30-4963). The ‘other’ category had the largest proportion of studies with fewer than 100 individuals (22%; Table 1).

**Table 1:** Samples sizes used to parameterize matrix models in 52 studies of plant demography (N = 68 species total).

Life history	N	Mean±1 SD	Median	Range	Prop. using <100 plants	No sample size reported
perennial herb	28	747.27±1223.92	214.94	30-4963	0.12	4
shrub	9	573.15±480.02	302.62	162-1276	0	1
tree	16	1311.7±2040.72	575	91-6905	0.09	5
other	15	584.81±558.84	362.5	71-1561	0.22	6

NSS is the number of studies that did not report the sample size used to parameterize models.

## Discussion

Our results demonstrate that biased estimates of λ can result from small sample sizes, as predicted by Jensen's Inequality. This is not because estimates of vital rates based on small sample sizes are biased. Rather, it is because small sample sizes can act in concert with low survival rates to increase sampling variation. This increase, combined with the nonlinear relationship between λ and the vital rates, results in an overestimation of the population growth rate. However our simulations also demonstrate that these biases rapidly become negligible with increasing sample sizes and as survival increases. The precise sample size at which bias diminishes will obviously vary between species, and additional studies with other demographic datasets are needed to evaluate the generality of these results. However, because *Heliconia acuminata*'s population and elasticity structure is common to many long-lived plants [27], [28], we believe the qualitative conclusions of our study will apply to other perennial plant species.

Interestingly, we also found the magnitude of the bias in λˆ was influenced by the structure of the population being sampled. The increase in bias observed when sampling with the more realistic “inverse J” distribution has important implications or the design of demographic studies. Using a novel analytical approximation, Gross [6] found that sampling more intensively those stages to which λ was more sensitive increased the precision of estimates of λ. In contrast, Münzbergová and Ehrlén [7] conducted simulation studies based on published demographic data and concluded that sampling equal numbers of individuals from different size class generally provided the most precise estimates. Although our simulations were not designed to resolve this seeming contradiction, we do note that *H. acuminata* population growth is especially sensitive to changes in the survival of individuals in the larger stage classes while being relatively robust to changes in fecundity [24]. Sampling from a population using the more realistic inverse J-distribution therefore increased the sampling variance of the demographically ‘important’ vital rates (e.g., survivorship of larger plants) and decreased the sampling variance of the less ‘important’ vital rates. Hence, our results suggest that sampling more individuals from stages whose vital rates have larger elasticity values–as recommended by Gross [6]–may not only increase the precision of estimates of λ, it may also increase their accuracy sensu [29].

In light of our results, it appears that most studies of plant demography we reviewed have sample sizes large enough to overcome potential biases resulting from sampling variance. However, there are clearly cases in which small sample sizes are unavoidable, most notably those in which species are elusive e.g., [30] or rare e.g., [31]. In these cases, researchers may benefit from modeling vital rates to improve precision of vital rate estimates [32], [33] or using data from closely related species [5], [34].

Despite the widespread use of matrix models in ecology and conservation, studies evaluating alternative sampling designs remain limited. Our results suggest that for many of the sample sizes used in demographic studies (Appendix S1), matrix models are probably robust to the biases resulting from sampling variance of vital rates. However, this conclusion may depend on the structure of populations or the distribution of sampling effort in ways that remain to be explored. We believe that the framework developed by Doak et al. [5] provides a powerful tool with which to identify the threshold at which biases become negligible and aid in the development of appropriate sampling protocols for matrix models. In addition, biases due to sampling variance could potentially be eliminated entirely by using “Integral Projection Models” [35], [36] to analyze demography, although we know of no studies have evaluated this possibility. Finally, we were surprised to find that 24% of the studies we reviewed failed to report the sample sizes on which their demographic models were based. We end with a call to researchers using matrix models to report the number of individuals used to parameterize the different stage classes of their models–basic information without which it is impossible to evaluate if and how the results of ecological studies are biased.

## Materials and Methods

Simulation models used to estimate how the accuracy of projections of λ varied with sample size were based on data collected during a long-term and large-scale study of plant demography conducted at Brazil's Biological Dynamics of Forest Fragments Project (BDFFP; 2°30′S, 60°W). The focal species for this study was *Heliconia acuminata*, a perennial herb native to central Amazonia and the Guyanas [37]. Descriptions of the study site and experimental design can be found elsewhere [24], [25], [38]. Briefly, permanent 50 m×100 m plots were established in 13 of the BDFFP's reserves in January 1998. All *H. acuminata* in each plot were marked and mapped and the number of vegetative shoots each plant had was recorded [24]. Since their establishment the plots have been surveyed annually to record plant growth, mortality, and the emergence of new seedlings (i.e., established plants less than 1 year old). The plots were also surveyed during the flowering season to record the identity of reproductive individuals. The analysis presented here is based on summary data from the 1998–2003 surveys conducted in six continuous forest plots; during this time period we marked, measured, and recorded the fates of N = 3842 plants in these sites.

**Figure 2:**
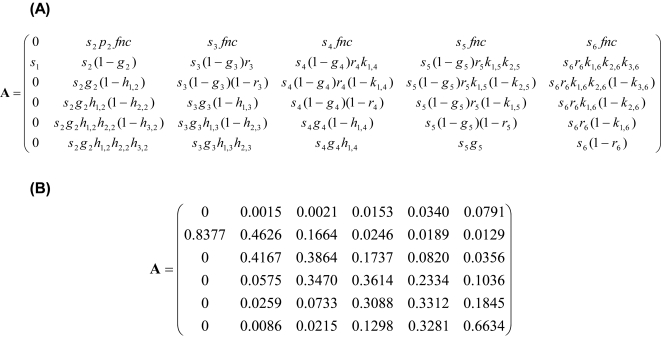
(A) *Heliconia acuminata* transition matrix used in Monte Carlo sampling analysis. The vital rates which compose each matrix element are defined as follows: *s_i_* = Prob(individual in stage *i* survives one time step), *g_i_* = Prob(individual in stage *i* grows at least one stage in one time step | survival), *h_x,i_* = Prob(individual in stage *i* grows at least *x* stages | growth of at least *x*−1 stages), *r_i_* = Prob(individual in stage *i* regresses at least one stage per time step | survived and did not grow), *k_x,i_* = Prob(individual in stage *i* regresses at least *x* stages | regression of at least *x*−1 stages), *p_i_* = Prob(plant in stage *i* flowers), *f_i_* = mean number of fruits per flowering plant in stage *i*, *n* = mean number of seeds per fruit, *c* = Prob(seed germinates and establishes) (B) *Heliconia acuminata* transition matrix used in sampling simulations (see *Methods*).

The demography of *Heliconia acuminata* can be described by the matrix shown in Figure 2. Note that there is no seed bank–all seeds produced in year *t* either germinate and become seedlings or die in year *t*+1. Our simulations used Bruna's [24] 1998–1999 transition year estimates of the vital rates from the continuous forest populations to calculate the ‘true’ population growth rate (i.e., λ); the results of our simulations were not sensitive to the choice of data to use as the reference year (results not shown).

We then simulated estimating λˆ for subsamples of the population ranging from 25–200 individuals. To do so, we used one of two probability distributions to simulate sampling each vital rate: a beta probability distribution for the binomial vital rates (e.g., probability of survival, probability of growth) and the gamma distribution for the count-based vital rates (i.e., fecundity). Because the beta distribution is continuous, bounded by 0 and 1, and can be parameterized to have a variety of means and variances, it is an appropriate choice for modeling estimates of the binomial vital rates [4]. We chose the gamma distribution to model estimates of average fecundity because it is non-negative and can also be flexibly parameterized. We parameterized both the beta and gamma sampling distributions according to the method of moments, a technique which parameterizes a distribution by specifying its expected value and variance [39]. To define the sampling process, we set the expected value and variance of an estimated vital rate equal to the population's mean vital rate and sampling variance, respectively. We determined the sampling variance at each sample size with equation (1) and equation (2). Then, we used well-known method of moments relationships between the parameters of the distributions and their expected value and variance e.g., [40]. For the beta distribution, we used the following relationship between the parameters and the mean and variance of the distribution:
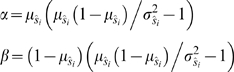
(3)where and *μ_ŝi_* and *σ_ŝi_* ^2^ are the mean and variance of the estimate of vital rate *s_i_*, respectively. Similarly, to calculate the parameters of the gamma distribution, we used the relationship:
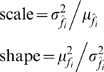
(4)where 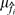 and 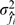 are the mean and variance of the estimate of fecundity *f_i_*, respectively. According to equation (1), the variance of binomial vital rate estimates is maximized when the rate is 0.5. To test for a difference in bias when survival estimates vary maximally, we simulated with both the true survival rates and after replacing the survival of all stages with 0.5. We also simulated at an intermediate survival level of 0.8, which is a high but realistic probability of individual mortality (IJF, *unpublished data*). Similarly, because the variance of estimates of fecundity is proportional to the real variance of fecundity among individuals (equation (1), we simulated with 3 levels of fecundity variance. We defined these levels in terms of coefficient of variation, 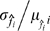: 0.5, 2, and 16.

We used two different population structures to conduct our simulations: a uniform distribution of individuals among size classes (i.e., equal numbers of individuals in all size classes) and the inverse J distribution [24], [26], in which there are fewer stage *i*+1 individuals than stage *i*. We used the actual distribution of classes observed in the field averaged over all years and sites to compute the inverse J distribution.

For each vital rate, we ran 2000 simulations with populations ranging in size from 25 to 200 individuals for all combinations of survival (the mean values from the *H. acuminata* demographic survey, henceforth called the “real” values, or mean survival = 0.5), fecundity (CV = 0.5, 2, or 16), and sampling distribution (an “inverse J” distribution or a “uniform” distribution). We chose 25 individuals as the smallest sample size because smaller samples would yield too few individuals per stage class to resemble what a real study might sample. In each run of the simulation, we drew all 31 vital rates (26 binomial vital rates and 5 fecundities) from their appropriate sampling distributions, computed a sample transition matrix from these vital rates, and then estimated λ as the dominant eigenvalue of this transition matrix (Figure 2).

We then estimated the expected value and standard deviation of the relative bias of λ estimates at each combination of survival rates, fecundity CV, distribution of sampling effort, and sample size. We calculated the relative bias as (λˆ−λ)/λ×100% where λ is the “true” asymptotic population growth rate and λˆ is the mean of all 2000 population growth rates estimated. All simulations were conducted using the R statistical computing environment [41].

To contextualize the results of our simulations, we conducted a review the plant demographic literature to determine the sample sizes used to parameterize matrix models. We conducted our survey using a Web of Science search from March 15, 2006. Our search terms were combinations of “matrix model”, “plant”, and “demography.” For each paper returned in our search, we used the “times cited” and “references cited” features to find additional relevant studies. For each study we identified the number of individuals sampled to parameterize non-reproductive terms of the matrix; if a study included more than one matrix (e.g., in multi-site or multi-year studies), we calculated the average number of individuals used.

## Supporting Information

Appendix S1 Studies using matrix models to study plant demography.
(0.11 MB PDF)
