# Supplementary material for: Effects of Sample Size on Estimates of Population Growth Rates Calculated with Matrix Models
Source: PLoS One. 2008 Aug 28;3(8):e3080. doi: 10.1371/journal.pone.0003080 (PMC2518208; doi:10.1371/journal.pone.0003080)
Supplement: Appendix S1 — Studies using matrix models to study plant demography. (0.11 MB PDF) [file pone.0003080.s001.pdf]

Appendix 1: Studies using matrix models to study plant demography. We used these studies to determine the sample sizes used to parameterize models.

1. Enright N, Ogden J (1979) Applications of transition matrix models in forest dynamics: *Araucaria* in Papua New Guinea and *Nothofagus* in New Zealand. Australian Journal of Ecology 4: 3-24.

2. Bullock SH (1980) Demography of an undergrowth palm in littoral Cameroon. Biotropica 12: 247-255.

3. Bierzychudek P (1982) The demography of Jack-in-the-Pulpit, a forest perennial that changes sex. Ecological Monographs 52: 335-351.

4. Meagher TR (1982) The population biology of *Chamaelirium luteum*, a dioecious member of the lily family 2. Sex population projections and stable population structure. Ecology 63: 1701-1711.

5. Lefebvre C, Chandler-Mortimer A (1984) Demographic characteristics of the perennial herb *Armeria maritima* on zinc lead mine wastes. Journal of Applied Ecology 21: 255-264.

6. Pinero D, Martinezramos M, Sarukhan J (1984) A Population model of *Astrocaryum mexicanum* and a sensitivity analysis of its finite rate of increase. Journal of Ecology 72: 977-991.

7. Fiedler PL (1987) Life history and population dynamics of rare and common Mariposa Lilies (*Calochortus pursh*: Liliaceae). Journal of Ecology 75: 977-996.

8. Huenneke LF, Marks PL (1987) Stem dynamics of the shrub *Alnus incana* ssp. *Rugosa*: transition matrix models. Ecology 68: 1234-1242.

9. Eriksson O (1988) Ramet behavior and population growth in the clonal herb *Potentilla anserina*. Journal of Ecology 76: 522-536.

10. Moloney KA (1988) Fine-scale spatial and temporal variation in the demography of a perennial bunchgrass. Ecology 69: 1588-1598.

11. Platt WJ, Evans GW, Rathbun SL (1988) The population dynamics of a long-lived conifer (*Pinus palustris*). American Naturalist 131: 491-525.

12. Charron D, Gagnon D (1991) The demography of northern populations of *Panax quinquefolium* (American Ginseng). Journal of Ecology 79: 431-445.

13. Nakashizuka T (1991) Population dynamics of coniferous and broad-leaved trees in a Japanese temperate mixed forest. Journal of Vegetation Science 2: 413-418.

14. Enright NJ, Watson AD (1992) Population dynamics of the Nikau Palm *Rhopalostylis sapida* (Wendl. et. Drude) in a temperate forest remnant near Auckland, New Zealand. New Zealand Journal of Botany 30: 29-43.

15. Calvo RN (1993) Evolutionary demography of orchids: intensity and frequency of pollination and the cost of fruiting. Ecology 74: 1033-1042.

16. Cipollini ML, Whigham DF, O'Neill J (1993) Population growth, structure, and seed dispersal in the understory herb *Cynoglossum virginianum*: a population and patch dynamics model. Plant Species Biology 8: 117-129.

17. Pinard M (1993) Impacts of stem harvesting on populations of *Iriartea deltoidea* (Palmae) in an extractive reserve in Acre, Brazil. Biotropica 25: 2-14.

18. Svensson BM, Carlsson BA, Karlsson PS, Nordell KO (1993) Comparative long-term demography of 3 species of *Pinguicula*. Journal of Ecology 81: 635-645.

19. Alvarez-Buylla ER (1994) Density dependence and patch dynamics in tropical rain forests: matrix models and applications to a tree species. American Naturalist 143: 155-191.

20. Cipollini ML, Wallacesenft DA, Whigham DF (1994) A model of patch dynamics, seed dispersal, and sex-ratio in the dioecious shrub *Lindera benzoin* (Lauraceae). Journal of Ecology 82: 621-633.

21. Ehrlen J (1995) Demography of the perennial herb *Lathyrus vernus*: II. Herbivory and population dynamics. Journal of Ecology 83: 297-308.

22. Horvitz CC, Schemske DW (1995) Spatiotemporal variation in demographic transitions of a tropical understory herb: projection matrix analysis. Ecological Monographs 65: 155-192.

23. Lesica P (1995) Demography of *Astragalus scaphoides* and effects of herbivory on population growth. Great Basin Naturalist 55: 142-150.

24. Olmsted I, Alvarez-Buylla ER (1995) Sustainable harvesting of tropical trees: demography and matrix models of two palm species in Mexico. Ecological Applications 5: 484-500.

25. Oostermeijer JGB, Brugman ML, deboer ER, dennijs HCM (1996) Temporal and spatial variation in the demography of *Gentiana pneumonanthe*, a rare perennial herb. Journal of Ecology 84: 153-166.

26. Allphin L, Harper KT (1997) Demography and life history characteristics of the rare Kachina daisy (*Erigeron kachinensis*, Asteraceae). American Midland Naturalist 138: 109-120.

27. Byers DL, Meagher TR (1997) A comparison of demographic characteristics in a rare and a common species of Eupatorium. Ecological Applications 7: 519-530.

28. Vavrek MC, mcgraw JB, Yang HS (1997) Within-population variation in demography of *Taraxacum officinale*: season- and size-dependent survival, growth and reproduction. Journal of Ecology 85: 277-287.

29. Batista WB, Platt WJ, Macchiavelli RE (1998) Demography of a shade-tolerant tree (*Fagus grandifolia*) in a hurricane-disturbed forest. Ecology 79: 38-53.

30. Valverde T, Silvertown J (1998) Variation in the demography of a woodland understorey herb (*Primula vulgaris*) along the forest regeneration cycle: projection matrix analysis. Journal of Ecology 86: 545-562.

31. Hoffmann WA (1999) Fire and population dynamics of woody plants in a neotropical savanna: matrix model projections. Ecology 80: 1354-1369.

32. Silva Matos DMS, Freckleton RP, Watkinson AR (1999) The role of density dependence in the population dynamics of a tropical palm. Ecology 80: 2635-2650.

33. Barot S, Gignoux J, Vuattoux R, Legendre S (2000) Demography of a savanna palm tree in Ivory Coast (Lamto): population persistence and life-history. Journal of Tropical Ecology 16: 637-655.

34. Guardia R, Raventos J, Caswell H (2000) Spatial growth and population dynamics of a perennial tussock grass (*Achnatherum calamagrostis*) in a badland area. Journal of Ecology 88: 950-963.

35. Matos JF, Trevisan MC, Estrada CA, Monasterio M (2000) Comparative demography of two giant caulescent rosettes (*Espeletia timotensis* and *E. spicata*) from the high tropical Andes. Global Ecology and Biogeography 9: 403-413.

36. Parker IM (2000) Invasion dynamics of *Cytisus scoparius*: a matrix model approach. Ecological Applications 10: 726-743.

37. Brewer JS (2001) A demographic analysis of fire-stimulated seedling establishment of *Sarracenia alata* (Sarraceniaceae). American Journal of Botany 88: 1250-1257.

38. Tolvanen A, Schroderus J, Henry GHR (2001) Demography of three dominant sedges under contrasting grazing regimes in the High Arctic. Journal of Vegetation Science 12: 659-670.

39. Tolvanen A, Schroderus J, Henry GHR (2001) Age- and stage-based bud demography of Salix arctica under contrasting muskox grazing pressure in the High Arctic. Evolutionary Ecology 15: 443-462.

40. Berg H (2002) Population dynamics in *Oxalis acetosella*: the significance of sexual reproduction in a clonal, cleistogamous forest herb. Ecography 25: 233-243.

41. Kiviniemi K (2002) Population dynamics of *Agrimonia eupatoria* and *Geum rivale*, two perennial grassland species. Plant Ecology 159: 153-169.

42. McCauley RA, Ungar IA (2002) Demographic analysis of a disjunct population of *Froelichia floridana* in the mid-Ohio River Valley. Restoration Ecology 10: 348-361.

43. Pico FX, Riba M (2002) Regional-scale demography of *Ramonda myconi*: remnant population dynamics in a preglacial relict species. Plant Ecology 161: 1-13.

44. Rae JG, Ebert TA (2002) Demography of the endangered fragrant prickly apple cactus, *Harrisia fragrans*. International Journal of Plant Sciences 163: 631-640.

45. Bruna EM (2003) Are plant populations in fragmented habitats recruitment limited? Tests with an Amazonian herb. Ecology 84: 932-947.

46. Garcia MB (2003) Demographic viability of a relict population of the critically endangered plant *Borderea chouardii*. Conservation Biology 17: 1672-1680.

47. Guedje NM, Lejoly J, Nkongmeneck BA, Jonkers WBJ (2003) Population dynamics of *Garcinia lucida* (Clusiaceae) in Cameroonian Atlantic forests. Forest Ecology and Management 177: 231-241.

48. Brys R, Jacquemyn H, Endels P, De Blust G, Hermy M (2004) The effects of grassland management on plant performance and demography in the perennial herb *Primula veris*. Journal of Applied Ecology 41: 1080-1091.

49. Endress BA, Gorchov DL, Noble RB (2004) Non-timber forest product extraction: Effects of harvest and browsing on an understory palm. Ecological Applications 14: 1139-1153.

50. Forbis TA, Doak DE (2004) Seedling establishment and life history trade-offs in alpine plants. American Journal of Botany 91: 1147-1153.

51. Hara M, Kanno H, Hirabuki Y, Takehara A (2004) Population dynamics of four understorey shrub species in beech forest. Journal of Vegetation Science 15: 475-484.

52. Menges ES, Quintana-Ascencio PF (2004) Population viability with fire in *Eryngium cuneifolium*: Deciphering a decade of demographic data. Ecological Monographs 74: 79-99.

53. Nordbakken JF, Rydgren K, Okland RH (2004) Demography and population dynamics of *Drosera anglica* and *D. rotundifolia*. Journal of Ecology 92: 110-121.

54. Stokes KE, Allchin AE, Bullock JM, Watkinson AR (2004) Population responses of Ulex shrubs to fire in a lowland heath community. Journal of Vegetation Science 15: 505-514.

55. Morris WF, Doak DF (2005) How general are the determinants of the stochastic population growth rate across nearby sites? Ecological Monographs 75: 119-137.
